# A new approach for modeling generalization gradients: a case for hierarchical models

**DOI:** 10.3389/fpsyg.2015.00652

**Published:** 2015-05-28

**Authors:** Koen Vanbrabant, Yannick Boddez, Philippe Verduyn, Merijn Mestdagh, Dirk Hermans, Filip Raes

**Affiliations:** Faculty of Psychology and Educational Sciences, University of LeuvenLeuven, Belgium

**Keywords:** stimulus generalization, repeated measures ANOVA, hierarchical (linear) models, individual differences, R, lme4

## Abstract

A case is made for the use of hierarchical models in the analysis of generalization gradients. Hierarchical models overcome several restrictions that are imposed by repeated measures analysis-of-variance (rANOVA), the default statistical method in current generalization research. More specifically, hierarchical models allow to include continuous independent variables and overcomes problematic assumptions such as sphericity. We focus on how generalization research can benefit from this added flexibility. In a simulation study we demonstrate the dominance of hierarchical models over rANOVA. In addition, we show the lack of efficiency of the Mauchly's sphericity test in sample sizes typical for generalization research, and confirm how violations of sphericity increase the probability of type I errors. A worked example of a hierarchical model is provided, with a specific emphasis on the interpretation of parameters relevant for generalization research.

## 1. Introduction

Conditioning can be defined as changes in behavior that result from regularities in the environment (De Houwer et al., 2013). Generalization helps to functionally adapt behavior, as it allows appropriate treatment of novel stimuli based on experience with related stimuli. Generalization occurs when a conditioned response (CR) is triggered by a stimulus that is different from the original conditional stimulus (CS; Vervliet et al., 2006). Guttman and Kalish (1956) were one of the first to investigate generalization after conditioning. They trained pigeons to peck at a colored key (i.e., conditioned stimulus; CS+). The frequency at which pigeons picked at keys of differed colors (i.e., generalization stimuli; GSs) was assessed after the learning task. They found that the response strength increased as a function of the similarity between the conditioned color and the test colors. This function is known as the generalization gradient (Shepard, 1965).

The current surge of renewed interest in generalization is largely motivated by its involvement in psychopathology. Generalization is, for example, the core aspect of what makes anxiety disorders so impairing: Fear does not remain specific to a single stimulus paired with danger but generalizes to a broad set of stimuli. A war veteran might, for instance, start to respond fearfully to anything remotely sounding like a gunshot, making life nothing short of unbearable (Dymond et al., 2014). Explaining individual differences in generalization is one of the central topics in this literature, because these differences presumably explain differences in the vulnerability to, and maintenance of psychopathology (Dymond et al., 2014). For example, Lissek et al. (2010) found more generalization in a group suffering from panic disorder compared to healthy controls. Lommen et al. (2010) found that individuals high in trait neuroticism generalized more than individuals low in trait neuroticism. Lenaert et al. (2012) demonstrated that overgeneral autobiographical memory is related to differences in generalized responding by showing that participants with limited memory specificity generalized more. It is clear that experimental and individual factors affect the generalization gradient.

In recent studies, generalization gradients are typically analyzed by means of repeated measures Analysis-of-Variance (rANOVA). However, rANOVA has several limitations for this type of data. In this article we therefore propose hierarchical models as an alternative for rANOVA, with a specific application for generalization research. The use of hierarchical models in psychology in general is not new and is already well documented (e.g., Baayen et al., 2008; Kliegl et al., 2010; Barr et al., 2013). We will highlight some of the limitations of rANOVA and discuss the relative strengths of hierarchical models. In a simulation we will demonstrate the drawbacks of rANOVA. After this simulation study a worked example of a hierarchical linear model is provided.

## 2. Repeated measurement models

### 2.1. Repeated measures analysis-of-variance

rANOVA is an extension of analysis-of-variance (ANOVA) and is used to compare groups on a variable that is measured repeatedly (Girden, 1992). In generalization research the (dependent) variable is measured repeatedly, because the responses are recorded over the full stimulus dimension (i.e., CS+ and GSs) for every subject. This stimulus dimension is included as a within-subject factor in the rANOVA model. The individual differences variable (e.g., high/low memory specificity, high/low neuroticism, diagnostic status, age, gender) is typically included as a between subjects factor (e.g., Lissek et al., 2008; Lommen et al., 2010; Dunsmoor et al., 2011; Lenaert et al., 2012) to investigate differences in generalized responding. Although rANOVA corrects for the repeated measurement nature of the data, it comes with several limitations. In the next paragraph we will discuss three limitations of rANOVA.

First, rANOVA handles the stimulus dimension as a factor with *n*-categorically different levels, with *n* referring to the number of stimuli included in the design. Treating the stimuli as categorically different is not always warranted. Shepard (1987) and Tenenbaum and Griffiths (2001), for example, state that the stimuli underlying generalization come from a continuous metric psychological space. Inspection of the stimuli used in generalization research gives support to this dimensional claim. Examples of often used stimuli are: different shades of gray (Lommen et al., 2010), circles that differ in circumference (Lissek et al., 2008), and morphs between two faces (Lenaert et al., 2012). Including the stimulus dimension as a categorical factor additionally brings about an excess use of degrees of freedom in the model. Therefore, from a statistical standpoint a continuous treatment is more parsimonious.

Second, rANOVA is inflexible in modeling and testing for continuous individual differences, because of the inability of rANOVA to handle continuous independent data. However, independent measures in generalization research are typically continuous or ordinal (e.g., neuroticism or autobiographical memory specificity scores). To conform to rANOVA these variables are transformed into a categorical variable. Categorizing a continuous predictor is advised against, especially in the case of an independent variable: this practice creates a severe loss of information, reduced power, and an increased probability of Type II errors (Maxwell and Delaney, 1993; Taylor and Yu, 2002; Royston et al., 2006). Including this categorical variable in interaction with the *n*-levels of the stimulus dimension, to test for group differences in generalization, leads to an excess growth of used degrees-of-freedom. On top of these technical considerations, the choice of an ideal cut-point for categorizing the continuous predictor can be subject to exploratory behavior, referred to as *researchers degree of freedom* by Simmons et al. (2011). This can lead to biased parameter estimates and erroneous conclusions.

Third, some of the assumptions that come with rANOVA are problematic for generalization research. Sphericity is the most important assumption. Sphericity refers to the situation where the variances of the differences between all pairs of stimuli are equal. This is generally interpreted as the demand of equal variances within the stimuli, and equal correlations between all stimulus pairs (Huynh and Feldt, 1970). Within generalization this implies that we assume that the relationship between all pairs of stimuli are equal. More specific, if we were to take each pair of stimuli from the used dimension and calculate difference scores between each pair, then it needs to hold that the variances of these pairs are equal. This assumption is unrealistic when analyzing generalization gradients for three reasons. First, sphericity is an unrealistic assumption for most repeated measures data. O'Brien and Kaiser (1985) claim that sphericity is commonly violated in most designs with more than two repeated measurement. Generalization studies often have multiple repeated measurements per subject. For example, Lenaert et al. (2012) used 8 different stimuli, Lissek et al. (2008) and Lommen et al. (2010) both use 10 different stimuli. The lowest value (i.e., the lower-bound estimate) a sphericity correction can adopt becomes smaller with an increasing number of stimuli. The lower the value, the stronger the sphericity correction will be. For example, the lower-bound correction with 10 stimuli is 1/(10−1) = 0.11, where every value smaller than 1 would indicate a sphericity violation. Second, because the CS+ and the CS− are training stimuli the variance around these stimuli is smaller than for example, a GS that lies in the middle of the continuum. Responses toward these GSs are more uncertain and will trigger more variability in responding across participants, see for example Figure 3 in Lenaert et al. (2012). Hence, the difference score between the CS+ and the CS− will be less variable than the difference score between the CS+ and a GS. Third, sphericity contradicts our understanding of how individual differences are manifested in generalization gradients: For example, we expect that subject with certain traits (i.e., low memory specificity, high anxiety, high neuroticism) will respond differently toward some stimuli (e.g., GSs close toward the CS+) but not so toward other stimuli. This will create patterns in the data that violate sphericity. As a result, the research question of interest in current generalization research implies a violation of sphericity. Violations of this assumption compromise the results of a rANOVA and will lead to inflated *F*-values for omnibus tests for main effects and interactions involving the within-subjects factor (Box, 1954; Huynh and Feldt, 1976). The use of rANOVA to analyze data that violates sphericity will use a Type I error rate that is higher than the suggested alpha level of the test (e.g., Hearne et al., 1983). Mauchly (1940) proposed a significance test for evaluating the sphericity assumption. A correction (e.g., Greenhouse and Geisser, 1959; Huynh and Feldt, 1976) is necessary to account for these inflated *F*-values when this test indicates a violation.

In the next section we will introduce the hierarchical framework with a focus on linear models. We will provide a short introduction and discuss how these models can overcome the limitations that are imposed by rANOVA in the context of generalization research.

### 2.2. Hierarchical linear model

Hierarchical Linear models (HLM) offer an alternative for the analysis of repeated measures data. The HLM framework uses the notion of levels to indicate clustering in the data. This clustering is caused by repeated measurements (i.e., level-1) for every subject (level-2) within generalization research. HLM is an extension of regular regression analysis where regression parameters are allowed common to all subjects (i.e., fixed effects) together with parameters that model these subject-specific deviations (i.e., random effects). These random effects account for clustering by explicitly modeling the individual differences (Verbeke and Molenberghs, 2009).

Because explaining individual differences in generalization is a central topic in the research literature about generalization, we immediately introduce a random intercept/random slope model that models the subject-specific gradient (i.e., individual differences in the generalization gradient). This model is given by:

(1) $$Y_{ij}=\beta_{0i}+\beta_{1i}d_{ij}+\epsilon_{ij}$$

and

(2) $$\beta_{0i}=\gamma_{00}+U_{0i}$$

(3) $$\beta_{1i}=\gamma_{10}+U_{1i}$$

Where *Y*_*ij*_ is the response strength of subject *i* on stimulus *j* from dimension *d*_*ij*_. The intercept and slope parameters are given by β_0*i*_ and β_1*i*_. The residuals on level-1, ϵ_*ij*_, are assumed to be normally distributed with a mean of 0 and variance σ^2^, ϵ_*ij*_ ~ 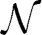
(0, σ^2^_ϵ_). The intercept β_0*j*_ consists of a fixed part, γ_00_, and a residual (i.e., random) part, *U*_0*i*_ at the subject level. The same holds for the slope parameter β_1*i*_: it consists of a fixed, γ_10_, and a residual (i.e., random) part, *U*_1*i*_. The residuals error on level-1 and level-2 are assumed to be independent. The residual errors on level-2 have a multivariate normal distribution. Their variance-covariance matrix is given by:

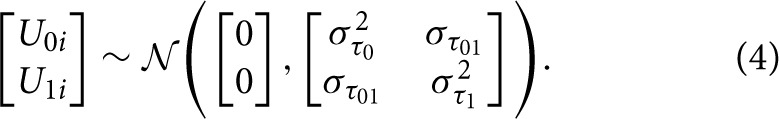



Where σ^2^_τ_0__ is the variance for the intercept, σ^2^_τ_1__ the variance for the slope, and σ_τ_01__ the covariance between the intercept and slope. We will continue with the discussion of four advantages of HLM for the analysis of generalization gradients.

First, HLM allows including the stimulus dimension as a continuous variable. As discussed earlier, treating the stimulus dimension as continuous is in line with theories of generalization. On top of this theoretical argument, this strategy has a technical advantage as well: Including the stimulus dimension as continuous variable opens the possibility to describe non-linear response patterns across the stimulus dimension (e.g., quadratic, cubic, logarithmic, exponential) in a parsimonious way (i.e., without using an excess of degrees of freedom). However, if theoretically warranted it is still possible to include the stimulus dimension as a factor with *n*-levels.

Second, hierarchical models are flexible with respect to modeling and testing individual differences. Including a variable, *u*_*i*_, that is measured at the subject level in Equations (2) and (3) leads to a model were individual differences in the intercept and slope can be explained. Formally these level-2 models are given by:

(5) $$b_{0i}=\gamma_{00}+\gamma_{01}u_{i}+U_{0i}$$

(6) $$b_{1i}=\gamma_{10}+\gamma_{11}u_{i}+U_{1i}$$

Where γ_01_ is the regression weight for *u*_*i*_ in the intercept model, and γ_11_ is the regression weight for *u*_*i*_ in the slope model. More specific, the γ_01_ parameter indicates the change in the intercept for a one unit change in *u*_*i*_ for subject *i*. This implicates that subjects who score higher/lower on *u*_*i*_ show a stronger/weaker response to the CS+.

The γ_11_ parameter indicates the change for the slope with a one unit increase of *u*_*i*_ for subject *i*. This implicates, assuming that the fixed slope effect γ_10_ is negative when modeling generalization gradients (i.e., decline in response strength over the dimension), a positive γ_11_ means that high scores on *u*_*i*_ have a less steep slope; a negative value for γ_11_ has the reverse interpretation. These interpretations demonstrate that γ_11_ is of special interest when modeling individual differences in the generalization gradient.

Third, the assumptions of HLM are generally the same as for standard regression models with the exception that observations do not need to be independent. The random effects account for this dependency. Violating the assumption of sphericity is of no concern in HLM, because the variance and covariance that cause sphericity are explicitly included in the model (Snijders and Bosker, 2012).

Fourth, hierarchical models can handle various data structures as dependent variable. For continuous dependent variables the HLM framework is suited. Generalized linear hierarchical models (GLHM) can offer a solution when the dependent variable is non-normally distributed. For example, the outcome of approach/avoidance tasks are binomial distributed and the GLHM can account for this through a link function. Different link functions in these GLHMs can account for various non-continuous data types (e.g., poisson link for count data, logit for binary data). For the interested reader, Tuerlinckx et al. (2006) give an extensive review of GLHMs.

In the next section we will use a simulation to compare the effectiveness of rANOVA and HLM for recovering a known generalization effect. Special attention is devoted to the sphericity assumption within rANOVA. We evaluate the effectiveness of Mauchly's sphericity test and demonstrate the effect of violations on the obtained results.

## 3. Simulation study

We conducted a simulation study to, in the first place, determine the influence of the dichotomizing process on the recovery rate of a true effect. For this reason we will compare a HLM that includes a continuous individual differences variable with a rANOVA that uses a dichotomized version of the original individual difference variable^1^. Second, given that sphericity is an unrealistic assumption in generalization research (see the third point in Section on Repeated Measures Analysis-of-Variance) we will to test how well Mauchly's sphericity test behaves in samples sizes typical for generalization research. It is already know that the Mauchly's test lacks power in small sample sizes. Afterwards we compare the results of an uncorrected rANOVA with a corrected ANOVA to inspect the differences in results when the violation of sphericity is ignored. The data was simulated according to a full random hierarchical model with the stimulus dimension as within subject factor. The dimension consisted of 10 stimuli. A between subject variable was simulated to account for the differences in generalization gradient. The simulation was performed using R software (RCoreTeam, 2014) version 3.1.1. The model can be found in Table 1 and computational details can be found in Appendix.

**Table 1:** **Details of the simulated data**.

**Parameter**	**Notation**	**Parameter value**
**FIXED PARAMETERS**		
Intercept	γ_00_	7.91871
Dimension	γ_10_	−0.58322
u	γ_01_	−0.37375
Dimension^*^u	γ_11_	[0.00, 0.05, 0.10]
**RANDOM PARAMETERS**		
Variance-covariance	$\left(\begin{array}{cc} \sigma_{\tau 0}^{2} & \sigma_{\tau 01}\\ \sigma_{\tau 01} & \sigma_{\tau 1}^{2} \end{array}\right)$	$\left(\begin{array}{cc} 2.5324 & -0.46\\ -0.46 & 0.1345 \end{array}\right)$
Within-participants	σ^2^_*e*_	2.4348

Two variables were manipulated in the simulation study: sample size and the effect size of the cross-level interaction γ_11_. The sample sizes were based on what we found in the generalization literature: *n* = 20 (Lissek et al., 2008), *n* = 38 (Lenaert et al., 2012), and *n* = 55 (Lommen et al., 2010). The size of γ_11_ was fixed at 0.00 (i.e., absence of effect), 0.05 (i.e., moderate effect), or 0.10 (i.e., large effect). Finally, the simulation was set to violate sphericity by sampling from an unstructured covariance matrix with a significant random slope-effect. All these manipulations combined led to 3 × 3 = 9 conditions.

Every condition made use of 1500 simulated samples. In the rANOVA model we were interested in the significance level of the interaction between the stimulus dimension and the dichotomized (based on a median split) individual differences variable, *u*_*i*_. In the HLM the parameter of the cross-level interaction between the stimulus dimension and the individual differences variable, γ_11_, was monitored. The output of both the rANOVA and the mixed model were compared against a α = 0.05 level. Mauchly's test of sphericity will be reported against a α = 0.05 level. We report the output from a rANOVA that is not corrected for sphericity violations and an rANOVA that is corrected. We followed the recommendations of Girden (1992) and used the Greenhouse-Geisser correction (Greenhouse and Geisser, 1959) instead of the lower-bound or Huyhn-Feldt correction (Huynh and Feldt, 1976). The Greenhouse-Geisser correction is less extreme than the lower-bound correction, but less liberal than the Huyhn-Feldt correction, and specifically suited for rather strong violations of sphericity or when there is no information available on sphericity. Of course, every repeated measurement design needs a proper evaluation of which correction is ideally suited. A comparison of sphericity corrections can be found in Collier et al. (1967).

The output of these simulations can be found in Table 2. In this table we report the proportion of significance for the total of 1500 simulations per condition.

**Table 2:** **Proportion of significance for sphericity-test at α = 0.05 and interaction test at α = 0.05 for rANOVA and HLM**.

***n***	**γ_11_**	**Sphericity-test**	**rANOVA**	**Corrected rANOVA**	**HLM**
20	0.00	0.745	0.089	0.043	0.033
20	0.05	0.771	0.195	0.121	0.278
20	0.10	0.959	0.999	0.995	1.000
38	0.00	0.987	0.107	0.051	0.034
38	0.05	0.991	0.451	0.328	0.521
38	0.10	0.999	0.869	0.776	0.950
55	0.00	1.000	0.083	0.046	0.031
55	0.05	0.953	0.365	0.261	0.449
55	0.10	1.000	0.909	0.845	0.985

Mauchly's Test for Sphericity was used. The corrected rANOVA made use of the Greenhouse-Geisser correction. The cross-level interaction for the hierarchical model were tested via a Wald-test.

First, we conclude from Table 2 that the Mauchly's sphericity test is not effective in small sample sizes. When the sample consisted of 20 individuals and there was no effect of the individual differences variable, only 75% of sphericity violations were flagged. Second, if the sphericity violation is ignored we clearly see an inflation of type I errors (i.e., when γ_11_ = 0.00 the acceptance rate of an effect was above the 0.05 level). Second, HLM outperformed rANOVA in all 9 conditions. When the true effect was absent (i.e., γ_11_ = 0.00) or large (i.e., γ_11_ = 0.10) the mixed model outperformed the rANOVA model, but the differences were small. The largest differences appeared when there was a moderate effect (i.e., γ_11_ = 0.05). The HLM was twice as effective as rANOVA when the effect sizes were moderate, independent of sample size. When taking sample size into account we demonstrated that both models benefited from a sample size larger than 20 subjects. Again, this shows that small sample sizes, even in experimental conditions, can hamper scientific progress (e.g., Button et al., 2013). All evidence together we can conclude that a hierarchical model clearly outperforms (even) a well-executed rANOVA with respect to recovering the true effect.

In the next part we will demonstrate through a worked example how a HLM can be used to analyze generalization data.

## 4. Worked example

### 4.1. Example data: generalization of social exclusion

The considered experimental dataset comes from an unpublished study conducted at the Center for the Psychology of Learning and Experimental Psychopathology. Subjects were recruited from a paid community sample. In total, 52 subjects (17 males) participated and their median age was 18 (range: 18–49). The aim of the study was to investigate if generalized responding could be observed toward new stimuli (i.e., GSs) that were close in similarity to a stimulus (i.e., CS+) that was continuously paired with feelings of social exclusion (i.e., unconditioned stimulus, US). Subjects played a game of Cyberball (Williams et al., 2000) against two other virtual players. In Cyberball, a ball is tossed around between three players: two virtual players and the subject. In our version of the game one virtual player (CS+) always excluded that subject from the game in order to induce feelings of exclusion (US). The second virtual player (CS−) tossed the ball at chance level toward the other virtual player or toward the subject. This acquisition phase consisted of 100 ball tosses. Five features characterized the virtual players: they were a student, which university they attended, their field of study, their major, and their minor. The CS+ and the CS− profile only overlapped at the highest level (i.e., both were students). In the generalization test, subjects were presented with 10 possible players (i.e., CS+, CS+, and 8 GSs) and had to indicate to what extent they expect that this specific player would exclude them if they would play a game of Cyberball. This US-expectancy was rated on a 10-point scale where 0 indicated *this player will not exclude me* and 10 indicated *this player will exclude me*. The GSs consisted of morphs between the CS+ and the CS− and decreased in similarity with the CS+ (and increased in similarity with the CS−). This experiment led to a normative generalization gradient, where strength of US-expectancy decreases with an increase in dissimilarity between the GS and the CS+. For didactic reasons, we need an individual difference variable that can explain the difference in the subject-specific generalization gradients, we created a variable, *u*, that ranges from 0 to 10 and is associated with the subject specific generalization function.

### 4.2. Hierarchical models

We fitted all models in R (RCoreTeam, 2014) by means of the lme4-package (Bates et al., 2013). We refer to Baayen (2008) for a general introduction to R (i.e., chapter 1) and for an extensive treatment of hierarchical data-analysis (i.e., chapter 7). Throughout this worked example we made use of maximum likelihood estimation. If the interest lies in a Bayesian approach Gelman et al. (2014) give a thorough theoretical introduction of Bayesian hierarchical models. Gelman and Hill (2006) (i.e., chapter 16 and 17) give a practical introduction on how to use BUGS/JAGS within R for the estimation of hierarchical models in a Bayesian framework.

#### 4.2.1. Hierarchical linear model

The simplest, useful hierarchical model that we can fit to this data is a random intercept model^2^ with a fixed effect of the stimulus dimension. The analysis starts with a call to the **lmer()** function of the lme4-package:


model1 = lmer(Expectancy ~ 1 + d + (1|ID),
     ↪ REML=FALSE, data=df)


This code starts with regressing the *Expectancy*-scores on the dimension, *d*. This part constitutes the fixed part of the model. The **(1|ID)** statement allows the intercept to vary over all subjects and controls for the repeated nature of the data. A “1” in R always indicates an intercept. The REML statement in the code controls the optimization procedure for the parameter estimates; Restricted Maximum Likelihood (REML) as well as Maximum Likelihood (ML) are provided in the package. By setting the REML statement to FALSE one chooses for ML estimates. For small sample sizes (i.e., *n* < 40) REML is preferred because it is an unbiased estimator. ML estimates are necessary when you want to compare two nested models with a different fixed model statement. REML assumes equivalent fixed effects between two competing nested models. Although ML is a biased estimator it behaves asymptotically unbiased in large sample sizes. In sum, if you have a small sample you need to use REML with the restriction that you cannot compare competing models with respect to their fixed effects. If your sample is large enough, you can use ML and benefit from the added flexibility (Snijders and Bosker, 2012). To end the model statement you need to provide a data frame that holds all the variables that are used in the model (for more information on data frames you can type **?data.frame** in R). The output of the model can be produced with the **summary (model1)** statement and is summarized in Table 3.

**Table 3:** **Output of the hierarchical linear models**.

	**Model 1**		**Model 2**		**Model 3**	
	**Parameter**	**S.E.**	**Parameter**	**S.E.**	**Parameter**	**S.E.**
γ_00_ = Intercept	7.92^***^	0.19	7.92^***^	0.28	9.79^***^	0.52
γ_10_ = Coefficient of d	−0.58^***^	0.03	−0.58^***^	0.06	−1.02^***^	0.12
γ_01_ = Coefficient of u					−0.44^***^	0.11
γ_11_ = Coefficient of d:u					0.10^***^	0.02
AIC	2253.26		2110.40		2098.56	
BIC	2270.28		2135.92		2132.59	
Deviance	2245.3		2098.4		2082.3	
Residual df	516		514		512	
Number of level-1 observation	520		520		520	
Number of level-2 clusters	52		52		52	
τ^2^_0_ = var(*U*_0*i*_)	0.37		3.33		2.33	
τ^2^_1_ = var(*U*_1*i*_)			0.18		0.13	
σ^2^_*e*_ = Var(ϵ_*ij*_)	4.12		2.43		2.43	

*** *p < 0.001; d, dimension; u, individual differences variable; U_0i_, random intercept effect; U_1i_, random slope effect; ϵ_ij_, level-1 residuals*.

The next model is the random intercept/random slope model which was formally introduced in Equation (1). The subject-specific generalization gradients are explicitly modeled by the added random slope effect. This model is specified as follows:


model2 = lmer(Expectancy ~ 1 + d + (1 + d|ID),
     ↪ REML=FALSE, data=df)


Only the random specification is altered in comparison with Model 1. The part **(1 + d|ID)** allows the intercept and slope to vary across subjects. To test the significance of random effects we need to introduce the notion of the Deviance statistic. This Deviance statistic indicates how well a model fits the data after controlling for the number of parameters included in the model (Gelman and Hill, 2006). In HLM this is mainly used to test the significance of random parameters. The difference in Deviance between two nested models is used to the test the effect of (an) added parameter(s). If the added random statement increases the fit of the model, a drop in Deviance will be observed that justifies added complexity of the model. This is done by comparing the random intercept/random slope model with the random intercept/fixed slope model. This comparison can be easily executed in R by means of the **anova()** statement:


anova(model1,model2)


which indicates a drop in Deviance of χ^2^_(2, 514)_ = 146.87, *p* < 0.01 after including the random slope effect. Note that the difference in Deviance is evaluated against two degrees of freedom, which come from the random slope parameter and the estimated covariance between the random intercept and random slope parameter. The Deviance statistic and parameter estimates of Model 1 and 2 can be found in Table 3. This (significant) difference indicates that there are individual differences in the degree of generalization across the stimulus dimension. A third model will be tested before we give an interpretation of the results.

This model includes an individual differences variable, *u*_*i*_, that can account for the variance around the intercept and slope. Including *u*_*i*_ as a main effect allows to explain variance at the level of the intercept. Including *u*_*i*_ in interaction with the stimulus dimension *d* allows to explain variance at the level of the slope. Especially this last parameter is of interest. The R-code:


model3 = lmer(Expectancy ~ 1 + d + u + d:u + (1
     ↪ + d|ID), REML=FALSE, data=df)


The output of this model is displayed in Table 3. Significance of fixed effects are evaluated by means of the Wald-test and are provided in the **lmer** output. The Intercept estimate indicates that the mean US-Expectancy for the CS+ is 9.79, after controlling for *u*_*i*_. The US-Expectancy for the CS+ decreases with 0.44 units for every unit increase in *u*. This means that high scores in *u* have a lower US-Expectancy for the CS+. The main effect of the stimulus dimension, *d*, indicates that every unit increase (e.g., going from GS2 to GS3) lowers the US-Expectancy toward that stimulus with 1.02 units. This observation corresponds to the definition of a generalization gradient: Responding to the GSs decrease as the difference with the original CS increases. The cross-level interaction between the stimulus dimension, *d*, and the individual difference variable, *u*, indicates that for every unit increase in *u* a reduction of 0.10 units is observed in the slope. This indicates that subjects who score higher on the individual difference variable will have a less steep slope, and hence, are generalizing more across the stimulus dimension. The reduction in slope variance between Model 3 and Model 2 is a direct effect of the cross-level interaction. We can see that 30% (from 0.18 to 0.13) of the slope variability is explained by including the individual difference variable.

**Figure 1:**
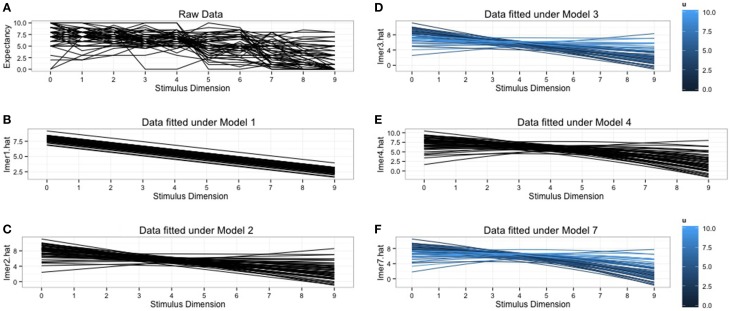
**Graphical representation of the raw data and the predicted data for the fitted models. (A)** The raw data plot for all 52 subjects. **(B)** Subjects specific predictions under the random intercept model. **(C)** Subject specific predictions under random intercept, random slope model. Notice the differences in slope for every subject. **(D)** Subject specific predictions from the random intercept, random slope model with inclusion of individual differences variable, *u*_*i*_. The shades of blue indicate the scores on *u*_*i*_, the lighter shades generalize more. **(E)** Subject specific predictions under the quadratic model. **(F)** Predictions under the quadratic model with inclusion of individual differences variable, *u*_*i*_. The shades of blue indicate the scores on *u*_*i*_, lighter shades generalize more.

We plotted the raw data, combined with the predicted data from the different models to get more insight in what the models are doing. These plots can be found in Figure 1. So far we always assumed a linear decrease in response strength. Figure 1A makes clear that this assumption is not realistic for the raw data. Clear deviations from this linear pattern can be detected. Polynomials can easily be introduced in the HLM framework when there is evidence that the generalization gradients deviate from linearity. In the next section we will give an example of how to fit a quadratic function without abandoning the **lmer**-function.

#### 4.2.2. Polynomial hierarchical linear model

The estimation of a polynomial function is analogous to a first order linear model. The regression weights in the models are still linear, only the stimulus dimension is included in a transformed version. The syntax will be similar to previously fitted models. We will start with extending Model 2 with a quadratic effect, *d*^2^, of the stimulus dimension. It is important to also include the first order effect when a quadratic effect is included. This fourth model is specified as follows:


model4 = lmer(Expectancy ~ 1 + d + I(d^2) + (1
     ↪ + d|ID), REML=FALSE, data=df)


In Table 4 you can see that the quadratic effect of the stimulus dimension significantly contributes to the fit of the model, *p* < 0.01. Alternatively the Deviance of Model 4 can be compared to Model 2 to test the quadratic effect. The difference in deviance is χ^2^_(1, 513)_ = 35.439, *p* < 0.01. An intermediate conclusion is that the generalization gradient is better described via a quadratic effect that allows some curvature over the stimulus dimension. Figure 1E demonstrates how this quadratic effect changes the fit of the model. In a next step we add a quadratic random effect. The fixed effect specification in R is the same as in Model 4, only the random part is altered:


(1 + d + I(d^2)|ID)


**Table 4:** **Output for the polynomial hierarchical linear models**.

	**Model 4**		**Model 5**		**Model 6**		**Model 7**	
	**Parameter**	**S.E.**	**Parameter**	**S.E.**	**Parameter**	**S.E.**	**Parameter**	**S.E.**
γ00 = Intercept	7.26^***^	0.30	7.26^***^	0.32	8.64^***^	0.60	8.69^***^	0.57
γ_10_ = Coefficient of d	−0.09	0.10	−0.09	0.12	−0.47^*^	0.21	−0.51^***^	0.14
γ_20_ = Coefficient of *d*^2^	−0.05^***^	0.01	−0.05^***^	0.01	−0.06^**^	0.02	−0.05^***^	0.01
γ_01_ = Coefficient of u					−0.28^**^	0.11	−0.29^**^	0.10
γ_11_ = Coefficient of d:u					0.08^*^	0.04	0.09^***^	0.02
γ_21_ = Coefficient of u:*d*^2^					0.00	0.00		
AIC	2076.96		2076.73		2066.84		2064.90	
BIC	2106.73		2119.27		2109.37		2103.18	
Deviance	2063.0		2056.7		2046.8		2046.9	
Residual df	513		510		510		511	
Number of level-1 observation	520		520		520		520	
Number of level-2 clusters	52		52		52		52	
τ^2^_0_ = var(*U*_0*i*_)	3.40		3.90		2.82		2.82	
τ^2^_1_ = var(*U*_1*i*_)	0.19		0.41		0.14		0.14	
τ^2^_2_ = var(*U*_2*i*_)			0.00					
σ^2^_*e*_ = Var(ϵ_*ij*_)	2.24		2.07		2.24		2.24	

*** p < 0.001,

** p < 0.01,

* p < 0.05;

*d, dimension; u, individual differences variable; U_0i_, random intercept effect; U_1i_, random slope effect of dimension; U_2i_, random slope effect of quadratic dimension; ϵ_ij_, level-1 residuals*.

The output from this model can be found in Table 4 under Model 5. The difference in Deviance between Model 4 and 5 is not significant, χ^2^_(3, 510)_ = 6.22, *p* = 0.10. This indicates that a random quadratic statement of *d* does not contribute to a better fitting model than a mere linear random effect.

We included *u* as a main effect and as a cross-level interaction with *d* and *d*^2^ to test if this individual difference variable explains the variability in the intercept and slope. This model is specified in R via:


model6 = lmer(Expectancy ~ 1+ d + u d:u +
     ↪ I(d^2) + I(d^2):u + (1 + d|ID),
     ↪ REML=FALSE, data=df)


An inspection of the output in Table 4 learns that the cross-level interaction between *d*^2^ and *u* is not significant (and also has no substantive contribution with parameters estimates around 0). Omitting this interaction leads to the final model. This model can be found under Model 7 in Table 4. As a substantive conclusion we can summaries that the generalization gradient is best described with a quadratic effect of the stimulus dimension. Second, the individual differences variable explains linear subject-specific deviations of this generalization gradient. In other words, the individual differences variable can predict the degree of generalization: High scores on the individual differences variable will have a more flattened generalization gradient.

Polynomials offer an elegant solution for fitting non-linear relationships within a linear framework. This example indicates that it can offer an interesting extension. However, polynomials come with some disadvantages. First, the interpretation of the model becomes more difficult because the first order and polynomial variable are highly correlated. Second, the interpretation of the polynomial takes place on a different scale: A one-unit increase needs to be interpreted on a non-linear scale. Third, one can easily over-fit the data when making use of higher order polynomials.

### 4.3. Comparison with repeated measures ANOVA

In this section we will analyze the data by means of rANOVA and compare the results with those obtained from the HLMs. In the current literature on generalization a continuous individual difference variable is dichotomized to fit into a rANOVA. Accordingly we will perform a median split on the individual difference variable *u*. This median split is given by:

(7) $${\text{Group}}_{i}=\{\begin{array}{ll} \text{low} & \;u_{i}<\text{median}(u)\\ \text{high} & \;u_{i}\geq \text{median}(u) \end{array}$$

Where Group_*i*_ refers to the group membership of subject *i*. This factor can take two levels: *low* when the value for *u* for subject *i* lies below the median for *u*, *high* when the value for *u* for subject *i* is equal to, or higher than the median for *u*. In a rANOVA model we included Group as a between-subject variable and Stimulus Type (CS+, CS−, and the GSs) as a within subject variable. Additionally, and of primary interest, the interaction between Group and Stimulus Type was included. Mauchly's test flagged that the assumption of sphericity was violated, *W* = 0.0003, *p* < 0.01. Therefore, we will report Greenhouse-Geisser ($\hat{\varepsilon }$ = 0.36) corrected tests. The results showed that there was no main effect of Group, *F*_(1, 50)_ = 0.80, *p* = 0.38, but there was a main effect of Stimulus Type, *F*_(3.28, 164.23)_ = 48.92, *p* < 0.01. The interaction effect between Group and Stimulus Type was also significant, *F*_(3.28, 164.23)_ = 6.32, *p* < 0.01. These results indicate that there is no difference in the overall US-Expectancy between the low and the high Group, but that different stimuli elicit a different mean US-expectancy. The interaction indicated that there are differences between the low and the high Group in the strength of response toward the different stimuli. This is exactly what was found with the HLM: There are differences in how subjects respond toward the different stimuli and (a dichotomized version of) *u* is meaningful in explaining these differences.

However, when we change the median split into:

(8) $${\text{Group}}_{i}=\{\begin{array}{ll} \text{low} & \;u_{i}\leq \text{median}(u)\\ \text{high} & \;u_{i}>\text{median}(u) \end{array}$$

the results of the rANOVA change drastically. Again Group_*i*_ refers to the group membership of subject *i*. This factor can take two levels: *low* when the value for *u* for subject *i* lies below or is equal to the median for *u*, *high* when the value for *u* for subject *i* is higher than the median for *u*. The same sphericity violation holds, we will again report Greenhouse-Geisser corrected tests. The results indicated no main effect of Group, *F*_(1, 50)_ = 1.66, *p* = 0.20 and a significant main effect of Stimulus Type, *F*_(3.28, 164.23)_ = 42.92, *p* < 0.01. However, the most important differences with the first rANOVA model lies in the absence of a significant interaction effect between Group and Stimulus type, *F*_(3.28, 164.23)_ = 1.402782, *p* = 0.24. This latter effect indicates that there are no differences between the two Groups in the strength of their responding toward the different stimuli. This is the opposite of what we found with the HLM.

These analyses make three points clear with respect to the use of rANOVA for generalization data. First, the sphericity violation is quite severe. In studies where there are 10 repeated measurements the lower limit of $\hat{\varepsilon }$ is 1/(10−1) = 0.11. The calculated value of $\hat{\varepsilon }$ in our study is 0.36 which is closer to the lower limit than toward the upper limit of 1. This means that quite severe corrections are in order to make correct inferences. Second, from the rANOVA output it is not possible to interpret the directions of the found effects (i.e., Which Group has a stronger overall US-expectancy? Which stimuli elicit the differences between groups?). Additional contrast analyses, and additional corrections from multiple testing, are in order to reach a level of interpretation that is necessary in generalization research. Third, the two performed rANOVAs indicated that a rather trivial change in the way in which a variable is dichotomized could alter the conclusions of the study drastically. This again indicates how problematic the dichotomization of a continuous independent variable.

## 5. Conclusion

In this paper we demonstrated that hierarchical models are superior for the analysis of generalization gradients. First, this superiority is mainly due to the possibility of using continuous independent variables in the model. Second, HLMs have fewer assumptions compared to rANOVA. Other than meeting the sphericity assumption it is essential for rANOVA to have complete data (i.e., complete case analysis). We tried to make a convincing case based on theoretical arguments and provided a simulation that demonstrated the true power of the hierarchical framework. In the last part of the paper a worked example was provided. In this tutorial a simple (i.e., linear) and more advanced (i.e., quadratic) models were introduced. This tutorial provided the appropriate R-code in order to apply HLM to new experimental data. We hope that this paper reaches it goal and can persuade all generalization researchers to use hierarchical models for the analysis of their data.

## Author note

The present article is supported by the KU Leuven Centre of Excellence on Generalization Research (GRIP^*^TT; PF/10/005) and in part by the Research Fund of KU Leuven (GOA/15/003) and by the Interuniversity Attraction Poles programme financed by the Belgian government (IAP/P7/06). The third author is a postdoctoral research fellow and the fourth author is a doctoral research fellow with the Fund for Scientific Research-Flanders (FWO).

### Conflict of interest statement

The authors declare that the research was conducted in the absence of any commercial or financial relationships that could be construed as a potential conflict of interest.

## Footnotes

^1^It would be possible to include the individual difference variable as a continuous covariate in a repeated measures analysis of covariance (rANCOVA). It is true that we are comparing two different variables: a dichotomized variable vs. a continuous variable. We do this because we want to compare current practice in generalization research, which a more flexible HLM approach. We intentionally did not discuss rANCOVA because this only offers a solution to one problem that arises when using rANOVA and we believe that HLM offer a more flexible and thorough approach.

^2^This model will be used as a basis for comparison of more complicated models.

## References

[B1] Baayen R. H. (2008). Analyzing Linguistic Data: A Practical Introduction to Statistics Using R. New York, NY: Cambridge University Press.

[B2] Baayen R. H. Davidson D. J. Bates D. M. (2008). Mixed-effects modeling with crossed random effects for subjects and items. J. Mem. Lang. 59, 390–412. 10.1016/j.jml.2007.12.005

[B3] Barr D. J. Levy R. Scheepers C. Tily H. J. (2013). Random effects structure for confirmatory hypothesis testing: keep it maximal. J. Mem. Lang. 68, 255–278. 10.1016/j.jml.2012.11.001 24403724 PMC3881361

[B4] Bates D. Maechler M. Bolker B. Walker S. (2013). lme4: Linear Mixed-Effects Models Using Eigen and S4. R Package Version 1.0-4 [Computer Software Manual]. Available online at: http://cran.r-project.org/web/packages/lme4/lme4.pdf

[B5] Box G. E. (1954). Some theorems on quadratic forms applied in the study of analysis of variance problems, i. effect of inequality of variance in the one-way classification. Ann. Math. Stat. 25, 290–302. 10.1214/aoms/1177728786

[B6] Button K. S. Ioannidis J. P. Mokrysz C. Nosek B. A. Flint J. Robinson E. S.. (2013). Power failure: why small sample size undermines the reliability of neuroscience. Nat. Rev. Neurosci. 14, 365–376. 10.1038/nrn3475 23571845

[B7] Collier R. O. Baker F. B. Mandeville G. K. Hayes T. F. (1967). Estimates of test size for several test procedures based on conventional variance ratios in the repeated measures design. Psychometrika 32, 339–353. 10.1007/BF02289596 5234710

[B8] De Houwer J. Barnes-Holmes D. Moors A. (2013). What is learning? on the nature and merits of a functional definition of learning. Psychon. Bull. Rev. 20, 631–642. 10.3758/s13423-013-0386-3 23359420

[B9] Dunsmoor J. E. Prince S. E. Murty V. P. Kragel P. A. LaBar K. S. (2011). Neurobehavioral mechanisms of human fear generalization. Neuroimage 55, 1878–1888. 10.1016/j.neuroimage.2011.01.041 21256233 PMC3062636

[B10] Dymond S. Dunsmoor J. E. Vervliet B. Roche B. Hermans D. (2014). Fear generalization in humans: Systematic review and implications for anxiety disorder research. Behav. Ther. 10.1016/j.beth.2014.10.001. (in press). 26459838

[B11] Gelman A. Carlin J. B. Stern H. S. Rubin D. B. (2014). Bayesian Data Analysis, Vol. 2. Boca Raton, FL: Taylor & Francis.

[B12] Gelman A. Hill J. (2006). Data Analysis Using Regression and Multilevel/Hierarchical Models. Cambridge: Cambridge University Press.

[B13] Girden E. R. (1992). ANOVA: Repeated Measures. Thousand Oaks, CA: Sage.

[B14] Greenhouse S. W. Geisser S. (1959). On methods in the analysis of profile data. Psychometrika 24, 95–112. 10.1007/BF02289823

[B15] Guttman N. Kalish H. I. (1956). Discriminability and stimulus generalization. J. Exp. Psychol. 51:79. 10.1037/h0046219 13286444

[B16] Hearne E. M. Clark G. M. Hatch J. P. (1983). A test for serial correlation in univariate repeated-measures analysis. Biometrics 39, 237–243. 6871352

[B17] Huynh H. Feldt L. S. (1970). Conditions under which mean square ratios in repeated measurements designs have exact f-distributions. J. Am. Stat. Assoc. 65, 1582–1589. 10.1080/01621459.1970.10481187

[B18] Huynh H. Feldt L. S. (1976). Estimation of the box correction for degrees of freedom from sample data in randomized block and split-plot designs. J. Educ. Behav. Stat. 1, 69–82. 10.3102/10769986001001069

[B19] Kliegl R. Masson M. E. Richter E. M. (2010). A linear mixed model analysis of masked repetition priming. Vis. Cogn. 18, 655–681. 10.1080/13506280902986058

[B20] Lenaert B. Claes S. Raes F. Boddez Y. Joos E. Vervliet B.. (2012). Generalization of conditioned responding: effects of autobiographical memory specificity. J. Behav. Ther. Exp. Psychiatry 43, 60–66. 10.1016/j.jbtep.2010.12.010 21237446

[B21] Lissek S. Biggs A. L. Rabin S. J. Cornwell B. R. Alvarez R. P. Pine D. S.. (2008). Generalization of conditioned fear-potentiated startle in humans: experimental validation and clinical relevance. Behav. Res. Ther. 46, 678–687. 10.1016/j.brat.2008.02.005 18394587 PMC2435484

[B22] Lissek S. Rabin S. Heller R. E. Lukenbaugh D. Geraci M. Pine D. S.. (2010). Overgeneralization of conditioned fear as a pathogenic marker of panic disorder. Am. J. Psychiatry 167, 47–55. 10.1176/appi.ajp.2009.09030410 19917595 PMC2806514

[B23] Lommen M. J. Engelhard I. M. van den Hout M. A. (2010). Neuroticism and avoidance of ambiguous stimuli: better safe than sorry? Pers. Individ. Dif. 49, 1001–1006. 10.1016/j.paid.2010.08.012

[B24] Mauchly J. W. (1940). Significance test for sphericity of a normal n-variate distribution. Ann. Math. Stat. 11, 204–209. 10.1214/aoms/1177731915

[B25] Maxwell S. E. Delaney H. D. (1993). Bivariate median splits and spurious statistical significance. Psychol. Bull. 113:181 10.1037/0033-2909.113.1.181

[B26] O'Brien R. G. Kaiser M. K. (1985). Manova method for analyzing repeated measures designs: an extensive primer. Psychol. Bull. 97:316. 10.1037/0033-2909.97.2.316 3983301

[B27] RCoreTeam (2014). R: A Language and Environment for Statistical Computing. Vienna: R Foundation for Statistical Computing.

[B28] Royston P. Altman D. G. Sauerbrei W. (2006). Dichotomizing continuous predictors in multiple regression: a bad idea. Stat. Med. 25, 127–141. 10.1002/sim.2331 16217841

[B29] Shepard R. N. (1965). Approximation to uniform gradients of generalization by monotone transformations of scale, in Stimulus generalization, ed Mostofsky D. (Stanford, CA: Stanford University Press), 94–110.

[B30] Shepard R. N. (1987). Toward a universal law of generalization for psychological science. Science 237, 1317–1323. 10.1126/science.3629243 3629243

[B31] Simmons J. P. Nelson L. D. Simonsohn U. (2011). False-positive psychology undisclosed flexibility in data collection and analysis allows presenting anything as significant. Psychol. Sci. 22, 1359–1366. 10.1177/0956797611417632 22006061

[B32] Snijders T. A. B. Bosker R. J. (2012). Multilevel Analysis: An Introduction to Basic and Advanced Multilevel Modeling. London: Sage.

[B33] Taylor J. M. Yu M. (2002). Bias and efficiency loss due to categorizing an explanatory variable. J. Multivariate Anal. 83, 248–263. 10.1006/jmva.2001.2045

[B34] Tenenbaum J. B. Griffiths T. L. (2001). Generalization, similarity, and bayesian inference. Behav. Brain Sci. 24, 629–640. 10.1017/S0140525X01000061 12048947

[B35] Tuerlinckx F. Rijmen F. Verbeke G. De Boeck P. (2006). Statistical inference in generalized linear mixed models: a review. Br. J. Math. Statist. Psychol. 59, 225–255. 10.1348/000711005X79857 17067411

[B36] Verbeke G. Molenberghs G. (2009). Linear Mixed Models for Longitudinal Data. New York, NY: Springer.

[B37] Vervliet B. Vansteenwegen D. Eelen P. (2006). Generalization gradients for acquisition and extinction in human contingency learning. Exp. Psychol. 53:132. 10.1027/1618-3169.53.2.132 16909938

[B38] Williams K. D. Cheung C. K. Choi W. (2000). Cyberostracism: effects of being ignored over the internet. J. Pers. Soc. Psychol. 79:748. 10.1037/0022-3514.79.5.748 11079239

