# Supplementary material for: A new approach for modeling generalization gradients: a case for hierarchical models
Source: Front Psychol. 2015 May 28;6:652. doi: 10.3389/fpsyg.2015.00652 (PMC4446539; doi:10.3389/fpsyg.2015.00652)
Supplement: Supplementary file 1 [file DataSheet1.PDF]

Supplementary Material for: A new approach for modeling generalization gradients: A case for Hierarchical Models

Koen Vanbrabant, Yannick Boddez, Philippe Verduyn, Merijn Mestdagh, Dirk Hermans,

Filip Raes

Faculty of Psychology and Educational Sciences, University of Leuven

Appendix: details of simulation

Required packages used for the simulation are: MASS (Ripley et al., 2014), lme4 (Bates et al., 2013), and ez (Lawrence, 2011). We wrote a function that that simulates a generalization dataset. The input parameters are: *n*, which manipulates the sample size (i.e., 20, 38, or 55); *effect* which manipulates the $\gamma_1 1$ (i.e., 0.00, 0.05, or 0.10). The last parameter, *n.sims* handles the amount of simulated data matrices. This parameter was always set to 1500 in this article. The function returns an R dataframe with 8 variables:

1. the estimate of $\gamma_1 1$ under the HLM
2. the standard error accompanying $\gamma_1 1$ under the HLM
3. the t-value for $\gamma_1 1$ under the HLM
4. F-value of the rANOVA
5. not-corrected p-value for the between and within subject interaction in the rANOVA
6. p-value of the Mauchly's sphericity test
7. Greenhouse-Geisser corrected p-value for the between and within subject interaction in the rANOVA
8. Huyhn-Feldt corrected p-value for the between and within subject interaction in the rANOVA

The function is given by:

```
###################
# Codes from Vanbrabant et al. A new approach for modeling
   ↪ generalization gradients: A case for Hierarchical Models
```

```
# Please contact Koen Vanbrabant
   ↪ (koen.vanbrabant@pw.kuleuven.be) with any questions
###################

# required packages
require(MASS)
require(lme4)
require(ez)

simulation = function(n,effect,n.sims){
  counter = 0
  ##########################################################################
  # preprare variables for out-put dataframe
  ##########################################################################
  mixed.estimate = rep(NA,n.sims)
  mixed.SE = rep(NA,n.sims)
  mixed.t = rep(NA,n.sims)
  f.value = rep(NA,n.sims) # F-value ANOVA
  p.value = rep(NA,n.sims) # p-value no-correction
  p.spher = rep(NA,n.sims) # p-value sphericity test
  p.GG = rep(NA,n.sims) # p-value after GG correction
  p.HF = rep(NA,n.sims) # p-value after HF correction
  association = data.frame(mixed.estimate,mixed.SE,mixed.t,
```

```
                              f.value,p.value,p.spher,p.GG,p.HF) #
                                 ↪ dataframe of association

    # loop for simulations
    for (j in 1:n.sims){
      n = n # [[Leneart (2012) = 38; Lommen (2011) = 55; Lissek
         ↪ (2008) = 20]]
      id = 1:n
      stim = 0:9
      id.long = as.factor(rep(id, each=length(stim)))
      stim.long = rep(stim,n)
      n.stim = length(stim)
      n.level1 = n*length(stim)
      range.level1 = 1:n.level1

      # values of fixed effecys
      g.00.true = 7.91871 # fixed intercept
      g.01.true = -0.37375 # intercept to put in interaction with
         ↪ u[j]

      g.10.true = -0.58322  # fixed slope
      g.11.true =  effect #  slope to put in interaction with u[j]

      u.sim = rnorm(n=n,mean=5,sd=3) # individual difference
         ↪ variable
```

```
u.max = max(u.sim)
u.min = min(u.sim)
u = round((u.sim-u.min)/(u.max-u.min)*10, digits=0)
u.long = rep(u,each=n.stim) # prepare individual differences
   ↪ for long dataframe

sigma.y = sqrt(2.4348) # level1 SD

d= matrix(c(2.5324,-0.46,-0.46,0.1345),2) #
   ↪ variance-covariance matrix
l = chol(d) # chol. decomposition
b0.true = rnorm(n,0,sd=l[1,1])
b1.true = rnorm(n)*l[1,2]+ rnorm(n,mean=0,sd=l[2,2])  #
   ↪ slope per ID

y.fake = rnorm(n.level1, # outcome data (e.g., US-Expectancy)
              g.00.true + g.01.true*u.long +
                 ↪ b0.true[id.long[range.level1]] + #
                 ↪ intercept
              (g.10.true + g.11.true*u.long +
                 ↪ b1.true[id.long[range.level1]])*stim.long
                 ↪  , # slope
              sd=sigma.y)
```

```
    df = data.frame(id.long,stim.long,y.fake,u.long) # make
       ↪ data.frame
    df$u.med = as.factor(ifelse(df$u.long <
       ↪ median(df$u.long),0,1))
    df$stim.factor = as.factor(stim.long)

### mixed model (ML)
mixedfit = lmer(y.fake ~ stim.long*u.long + (1 +
   ↪ stim.long|id.long), data=df,REML=FALSE,
                control=lmerControl(optimizer="bobyqa",
                optCtrl=list(maxfun=200000)))
summary(mixedfit)
    association[j,1] = coef(summary(mixedfit))[4,1] # estimate
    association[j,2] = coef(summary(mixedfit))[4,2] # SE
    association[j,3] = coef(summary(mixedfit))[4,3] # t-value

### rANOVA
rANOVA = ezANOVA(data = df,
                wid = .(id.long),
                dv = .(y.fake),
                within = .(stim.factor),
                between = .(u.med))
association[j,4] = rANOVA[[1]][3,4] # F-value
```

```
association[j,5] = rANOVA[[1]][3,5] # p-value no-correction
association[j,6] = rANOVA[[2]][2,3] # p-value sphericity test
association[j,7] = rANOVA[[3]][2,3] # p-value after GG correction
association[j,8] = rANOVA[[3]][2,6] # p-value after HF correction

counter = counter + 1 # counter to monitor progress
print(counter)
  }
return(association)
} # close function
```

The function was then called for all the 9 condition. For example

```
condition1 = simulation(20,0.00,1500) # sample size = 20,
   ↪ cross-level interaction = 0.00, and 1500 simulations were
   ↪ performed
```

Frequencies and proportions of significance were obtained via:

```
condition1$mixed.effect = ifelse(condition1$mixed.t > 1.96, 1,0)
   ↪ ; sum(condition1$mixed.effect) ;
   ↪ sum(condition1$mixed.effect)/1500
condition1$p.Mauchly = ifelse(condition1$p.spher < 0.05, 1, 0) ;
   ↪ sum(condition1$p.Mauchly) ; sum(condition1$p.Mauchly)/1500
condition1$p.GreenhouseGeisser = ifelse(sim1$p.GG < 0.05, 1, 0)
   ↪ ; sum(sim1$p.GreenhouseGeisser) ;
   ↪ sum(sim1$p.GreenhouseGeisser)/1500
```

```
condition1$p.HuyhnFeldt = ifelse(sim1$p.HF < 0.05, 1, 0) ;
    ↪ sum(sim1$p.HuyhnFeldt) ; sum(sim1$p.HuyhnFeldt)/1500 # not
    ↪ included in the paper.
condition1$p.nocorrection = ifelse(sim1$p.value < 0.05, 1,0) ;
    ↪ sum(sim1$p.nocorrection)
```
